# Predictive factors on postoperative venous thromboembolism after minimally invasive colorectal cancer surgery: a retrospective observational study

**DOI:** 10.1186/s12893-023-01992-x

**Published:** 2023-04-11

**Authors:** Dabin Wu, Haitao Gu, Yunhao Tang, Linglong Peng, Hang Liu, Yahui Jiang, Zhiquan Xu, Qi Wei, Yaxu Wang

**Affiliations:** Department of Gastrointestinal Surgery, The Second Affiliated Hospital of Chongqing Medical University, Chongqing, 400010 China

**Keywords:** Minimally invasive surgery, Colorectal cancer, Venous thromboembolism, Predictive factors

## Abstract

**Background:**

Venous thromboembolism (VTE) is a serious and preventable postoperative complication. However, the predictive significance of perioperative biochemical parameters for VTE after minimally invasive colorectal cancer surgery remains unclear.

**Methods:**

A total of 149 patients undergoing minimally invasive colorectal cancer surgery were collected between October 2021 and October 2022. Biochemical parameters related to preoperative and postoperative day 1, day 3, and day 5 were collected, including D-Dimer, mean platelet volume (MPV), and maximum amplitude (MA) of thromboelastography (TEG). Receiver operating characteristic (ROC) curves were used to explore the predictive powers of meaningful biochemical parameters for postoperative VTE, and calibration curves were used to assess predictive accuracy.

**Results:**

The overall cumulative incidence of VTE was 8.1% (12/149). The preoperative and postoperative day 3 D-Dimer, postoperative day 3, and day 5 MPV, and postoperative day 1, day 3, and day 5 TEG-MA was significantly higher in the VTE group than in the non-VTE group (*P* < 0.05). The results of both the ROC curve and the calibration curve indicated that these meaningful D-Dimer, MPV, and TEG-MA had moderate discrimination and consistency for postoperative VTE.

**Conclusions:**

D-Dimer, MPV, and TEG-MA may predict postoperative VTE in patients undergoing minimally invasive surgery for colorectal cancer at specific times in the perioperative period.

## Introduction

Globally, colorectal cancer (CRC) is one of the most common malignant neoplasms, and it poses a serious threat to human life and health [1]. Treatment for CRC is limited, and surgery combined with chemotherapy and radiotherapy has been considered a pivotal approach for patients with resectable tumors [2]. With the increasing development of technology, laparoscopic and robotic surgery has been widely used in the treatment of CRC patients. Laparoscopic and robotic surgery is known as minimally invasive surgery because it causes less trauma compared to traditional open surgery. However, regardless of the type of surgery, complications can occur after the surgery.

Venous thromboembolism (VTE) is the second leading cause of death among cancer patients undergoing medical and surgical treatment [3]. VTE is composed of deep vein thrombosis (DVT) and its complication pulmonary embolism (PE) [4, 5]. Due to the high risk of recurrent thromboembolism and bleeding, the treatment of venous thromboembolism is challenging in cancer patients [6]. Moreover, surgery is considered to have a proinflammatory effect on the occurrence of VTE [7]. The patient’s hypercoagulable state, venous blood pooling and vessel wall injury are all likely to occur during the surgery. Tissue factor exposure at the surgical site is also an important driver for the occurrence of VTE after surgery [8]. Previous studies have shown that red blood cell transfusions are associated with an increased risk of VTE in patients undergoing surgical resection of the colorectal [9]. Furthermore, platelets are essential for hemostasis and contribute to venous thrombosis through platelet G protein-coupled (GPCR) and immunoreceptor tyrosine-based activation motif (ITAM) receptor signaling [10]. It has been shown that platelet-related biochemical parameters are predictive of DVT in breast cancer patients [11], while thromboelastography (TEG)-related biochemical parameters are also predictive of VTE in gynecologic oncology patients [12]. However, previous studies lacked a focus on the effect of surgery on VTE and ignored the changes in biochemical parameters after surgery. The incidence of VTE events remains high in patients undergoing colorectal surgery [13, 14], and VTE also poses a serious threat to the health of cancer patients undergoing surgical treatment [3, 15]. Hence, there is a need to explore the predictive role of relevant biochemical parameters on the occurrence of postoperative VTE in patients with colorectal cancer treated with minimally invasive surgery.

In this study, we first compared the general data of VTE patients and non-VTE patients after minimally invasive colorectal cancer surgery. Next, we collected biochemical parameters related to the perioperative period in CRC patients. Finally, the predictive role of biochemical parameters on the occurrence of postoperative VTE was assessed by constructing ROC curves and calibration curves.

## Materials and methods

### Study Population

A retrospective observational study was conducted on consecutive patients who underwent minimally invasive colorectal cancer resection at the Second Hospital of Chongqing Medical University from October 2021 to October 2022. Inclusion criteria of the study: All patients had been diagnosed with colorectal adenocarcinoma by pathology preoperatively and no distant metastases were present; all patients underwent laparoscopic or robot surgery for colorectal cancer (All robotic surgery in this study were performed by the da Vinci Surgical System); all patients had no VTE confirmed by color Doppler ultrasound before the surgery. 202 patients were evaluated for inclusion in the study.

Exclusion criteria of the study: Patients who had received any thromboprophylaxis in the perioperative period (n = 24); patients with a previous history of thromboembolism (n = 1); patients who were converted to open surgery during the operation because minimally invasive surgery could not be performed (n = 2); patients with a previous history of malignancy of other organs (n = 3); patients with a previous history of preoperative chemotherapy or radiation therapy for malignancy (n = 15); patients who refuse to sign informed consent (n = 8). The final analysis included 149 patients. This study was approved by the Ethics Committee of the Second Hospital of Chongqing Medical University (Chongqing, China, Approval date: September 20, 2021, Ethical approval number: (2021) 507), and written informed consent was obtained from all patients.

### Biochemical parameters

D-dimer was a biomarker of fibrinolysis and coagulation and was analyzed using HemosIL D-dimer HS 500 (Lexington, MA, USA). Mean platelet volume (MPV) was a measure of platelet size and was a potential marker of platelet activation [16]. A whole blood autoanalyzer (Sysmex XN-3000, Kobe, Japan) was used to measure platelet count and MPV. Thromboelastography (TEG) is a widely used coagulation test that allows dynamic monitoring of the clotting response from fibrin formation to clot lysis [17]. The TEG was analyzed on TEG 5000 Thromboelastograph Analyzer (Haemonetics Corporation, MA, USA), and the parameters of TEG in this study included reaction time (R, min), solidification time (K, min), alpha angle (α, degrees), and maximum amplitude (MA, mm). D-Dimer, platelet count, MPV, and TEG were collected from patients’ preoperative and postoperative day 1, day 3, and day 5 through medical record data.

### Diagnosis of venous thromboembolism

Compression ultrasonography was performed according to standard procedures (grey scale, B-mode, color Doppler), and all VTE occurrences in this study were confirmed using a high-end ultrasound scanner (LOGIQ 9; GE, CA, USA) on day 8±2 after the surgery.

### Statistical analysis

Continuous variables were compared using Student’s t-test and presented as mean ± standard deviation (SD). Categorical variables were tested by chi-square (χ^2^) test and expressed as percentages. Receiver operating characteristic (ROC) curves were used to assess the discrimination of the venous thromboembolism prediction model, where the area under the curve (AUC) of the ROC was used to quantify. Calibration curves and the Hosmer-Lemeshow test were used to assess the calibration performance of the venous thromboembolism prediction model.

All statistical analyses were conducted with SPSS version 25.0 (IBM, Armonk, NY, USA). Two-tailed *P* values < 0.05 were considered statistically significant. A *P* value > 0.05 for the Hosmer-Lemeshow test was considered as having no significant difference between the predicted and observed events.

## Results

**Fig. 1:**
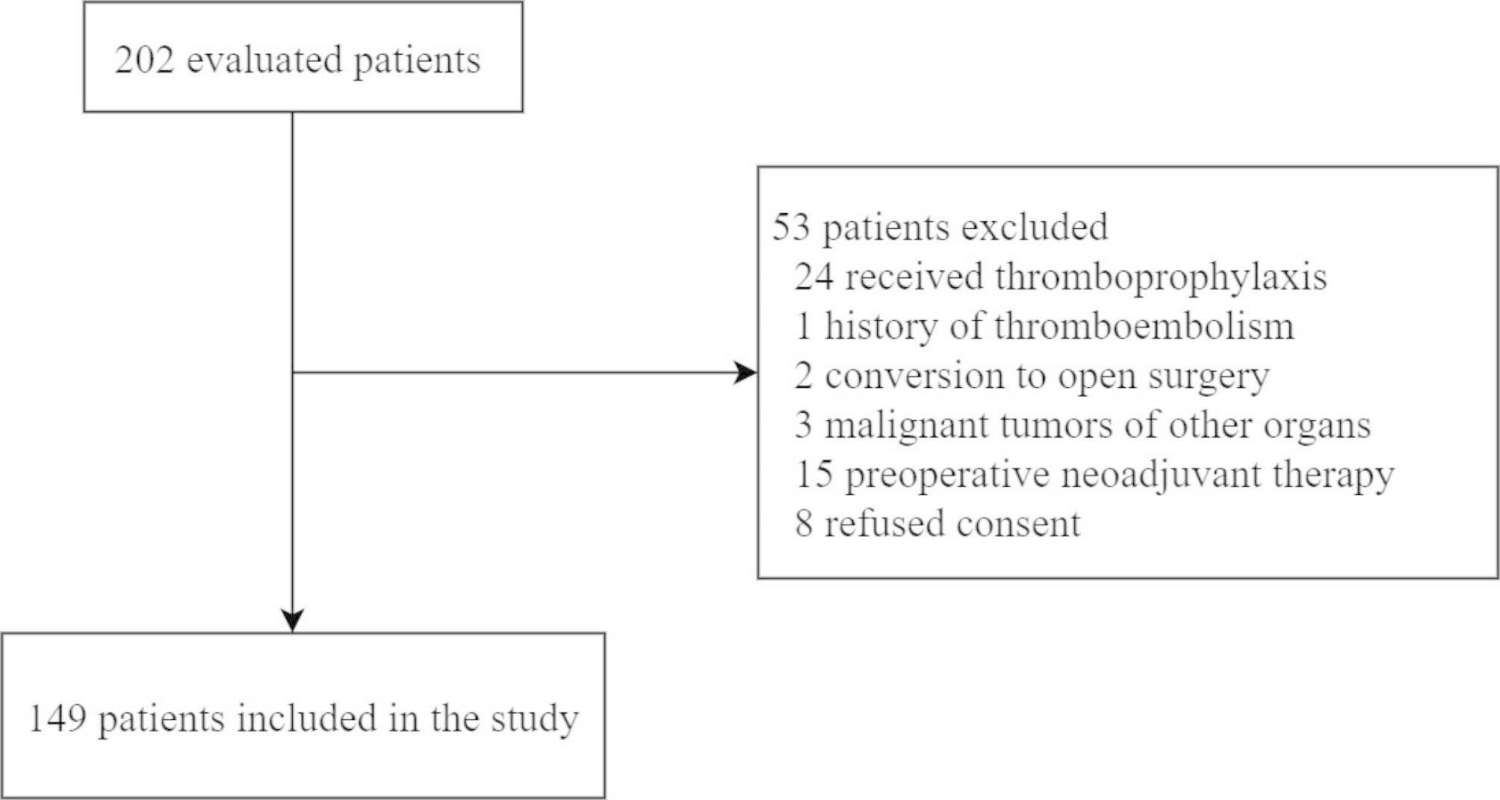
Flow chart of the study

Overall, 202 patients were evaluated and a total of 149 patients were included in this study (Fig. 1). Among them, 97 cases underwent laparoscopic surgery, and 52 cases underwent robotic surgery. As shown in Table 1, postoperative VTE occurred in 12 of 149 patients, with a cumulative outcome incidence of 8.1%. All these VTE patients occurred with DVT, and one had a secondary PE due to DVT (8.3%). The mean age at surgery for VTE patients was 69.58 years, which was higher than the mean age at surgery for non-VTE patients, which was 60.40 years, although they were not statistically different. As shown in Table 1, the occurrence of VTE after surgery was associated with an increase in ASA score (*P* = 0.012). 9 of 12 (75%) patients with VTE had an ASA score of 3. In this study, we did not find any correlation between the occurrence of postoperative VTE and other general information such as gender, BMI, smoking status, history of diabetes mellitus, cardiac disease, history of chronic renal failure, operation time, operative procedure, type of resection, and pathological stage of patients.

**Table 1:** General information for VTE

	VTE (n = 12)	No VTE (n = 137)	t value/χ2 value	P value
Sex (n, %)			0.100	0.752
Male	6 (50.0)	62 (45.3)		
Female	6 (50.0)	75 (54.7)		
Age (years)	69.58 ± 11.22	60.40 ± 12.51	-2.457	0.629
BMI (kg/m2)	22.91 ± 2.39	22.83 ± 2.42	-0.111	0.602
ASA (n, %)			6.262	**0.012**
2	3 (25.0)	85 (62.0)		
3	9 (75.0)	52 (38.0)		
Smoker (n, %)	3 (25.0)	16 (11.7)	1.760	0.185
Diabetes (n, %)	1 (8.3)	14 (10.2)	0.043	0.835
Cardiac disease (n, %)	1 (8.3)	3 (2.2)	1.594	0.207
History of chronic renal failure (n, %)	0 (0)	1 (0.7)	0.088	0.766
Operation time (min)	245.42 ± 67.60	231.01 ± 58.76	-0.805	0.223
Operative procedure (n, %)			0.263	0.608
Laparoscopic	7 (58.3)	90 (65.7)		
Robotic	5 (41.7)	47 (34.3)		
Type of resection (n, %)			0.396	0.983
Partial resection of the transverse colon	0 (0)	4 (2.9)		
Right hemicolectomy	1 (8.3)	11 (8.0)		
Left hemicolectomy	1 (8.3)	10 (7.3)		
Sigmoid colon resection	3 (25.0)	31 (22.6)		
Rectal resection	7 (58.4)	81 (59.2)		
Pathological Stages (n, %)			1.564	0.458
I	0 (0)	13 (9.5)		
II	6 (50.0)	72 (52.6)		
III	6 (50.0)	52 (37.9)		

VTE: venous thromboembolism, BMI: body mass index, ASA: American Society of Anesthesiology

In this study, we found that patients with VTE had significantly higher D-Dimer preoperatively and at postoperative day 3 than non-VTE patients (550.18 ± 357.71 vs. 279.58 ± 225.54, *P* = 0.028; 1564.77 ± 672.11 vs. 809.31 ± 443.35, *P* = 0.014). And MPV was significantly higher in the VTE patients than in the non-VTE patients at postoperative day 3 and day 5 (12.40 ± 1.85 vs. 10.58 ± 0.93, *P* < 0.001; 12.61 ± 1.43 vs. 10.56 ± 1.12, *P* = 0.046). Moreover, TEG-MA was significantly higher in the VTE patients than in the non-VTE patients at postoperative day 1, day 3, and day 5 (70.75 ± 0.90 vs. 68.81 ± 2.39, *P* = 0.015; 73.78 ± 3.90 vs. 70.21 ± 2.16, *P* = 0.021; 74.27 ± 2.69 vs. 71.24 ± 1.84, *P* = 0.029). However, no significant differences were seen in other relevant biochemical parameters (Table 2).

**Table 2:** Relevant biochemical parameters

	VTE (n = 12)	No VTE (n = 137)	t value	P value
D-Dimer (ng/mL)				
Pre-op	550.18 ± 357.71	279.58 ± 225.54	-3.777	**0.028**
D1	1319.98 ± 704.71	928.05 ± 523.76	-2.414	0.288
D3	1564.77 ± 672.11	809.31 ± 443.35	-5.404	**0.014**
D5	1787.64 ± 948.71	1063.90 ± 708.85	-3.295	0.127
Platelet (x 109/L)				
Pre-op	242.17 ± 45.84	225.75 ± 50.95	-1.078	0.823
D1	207.42 ± 57.79	209.05 ± 45.44	0.117	0.377
D3	200.08 ± 64.06	219.27 ± 46.92	1.316	0.089
D5	230.25 ± 47.78	219.74 ± 44.30	-0.783	0.588
MPV (fL)				
Pre-op	10.67 ± 0.88	10.37 ± 0.81	-1.206	0.644
D1	11.59 ± 1.07	10.56 ± 0.83	-4.044	0.209
D3	12.40 ± 1.85	10.58 ± 0.93	-5.876	**< 0.001**
D5	12.61 ± 1.43	10.56 ± 1.12	-5.935	**0.046**
R value (min)				
Pre-op	6.30 ± 0.81	6.66 ± 0.85	1.410	0.500
D1	6.32 ± 0.87	6.61 ± 0.82	1.149	0.866
D3	6.50 ± 0.78	6.65 ± 0.88	0.586	0.348
D5	6.49 ± 0.75	6.51 ± 0.77	0.083	0.743
K value (min)				
Pre-op	1.95 ± 0.67	2.04 ± 0.55	0.534	0.158
D1	2.01 ± 0.51	2.05 ± 0.53	-0.016	0.331
D3	2.01 ± 0.66	2.02 ± 0.59	0.045	0.528
D5	1.98 ± 0.60	2.07 ± 0.61	0.514	0.824
α angle (degree)				
Pre-op	62.82 ± 6.20	63.10 ± 5.71	0.157	0.736
D1	64.96 ± 5.37	62.69 ± 5.06	-1.480	0.795
D3	62.37 ± 4.57	63.15 ± 5.09	0.513	0.380
D5	60.82 ± 3.88	62.06 ± 4.80	0.869	0.230
MA (mm)				
Pre-op	64.00 ± 2.98	62.55 ± 4.15	-1.187	0.198
D1	70.75 ± 0.90	68.81 ± 2.39	-2.777	**0.015**
D3	73.78 ± 3.90	70.21 ± 2.16	-5.073	**0.021**
D5	74.27 ± 2.69	71.24 ± 1.84	-5.266	**0.029**

The *p*-values shown in bold are statistically significant (p < 0.05). MPV: mean platelet volume, MA: maximum amplitude of thromboelastography

**Fig. 2:**
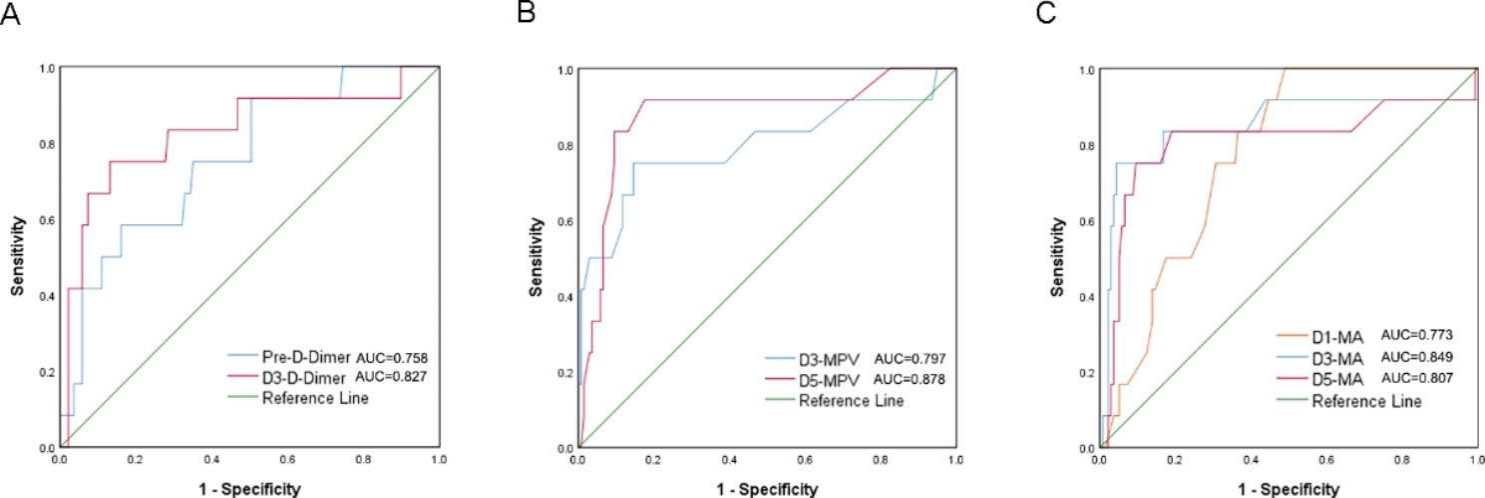
**Receiver operating characteristic (ROC) curves.** (A) D-Dimer at preoperative and postoperative day 3. (B) mean platelet volume (MPV) at postoperative day 3 and day 5. (C) maximum amplitude (MA) of thromboelastography at postoperative day 1, day 3, and day 5. AUC: area under the curve

**Fig. 3:**
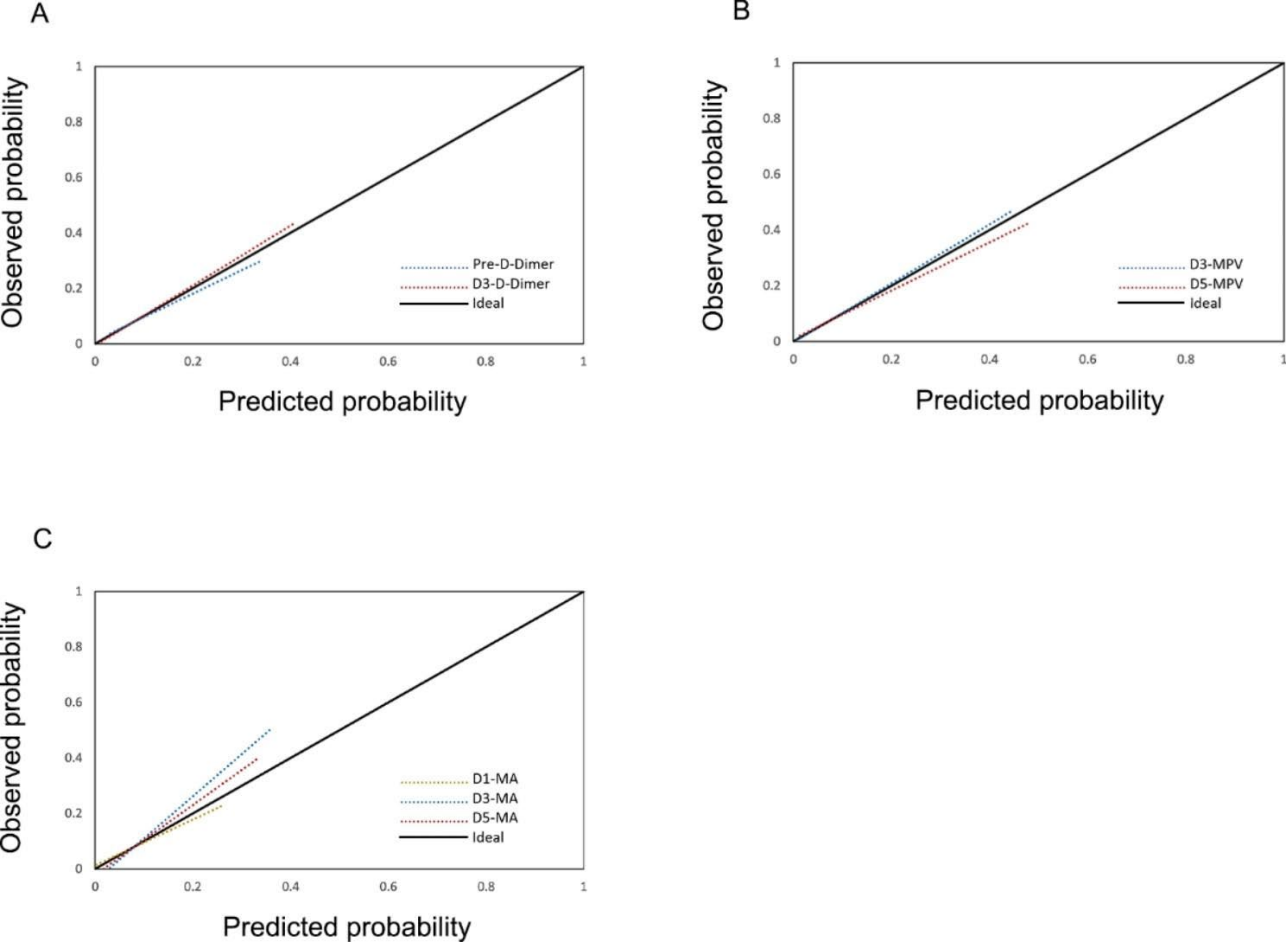
**Calibration Curve.** (A) D-Dimer at preoperative and postoperative day 3. (B) mean platelet volume (MPV) at postoperative day 3 and day 5. (C) maximum amplitude (MA) of thromboelastography at postoperative day 1, day 3, and day 5. All *P*-values > 0.05 for the Hosmer-Lemeshow test

The ROC curves were constructed to assess the discrimination of meaningful biochemical parameters in predicting postoperative venous thromboembolism. As shown in Fig. 2A, the ROC curve areas of D-Dimer preoperatively and postoperative day 3 were 0.758 and 0.827, respectively. The ROC curve areas of MPV at postoperative day 3 and day 5 were 0.797 and 0.878, respectively (Fig. 2B). As shown in Fig. 2C, the ROC curve area of TEG-MA was 0.773, 0.849, and 0.807 at postoperative day 1, day 3, and day 5, respectively. Based on the ROC curves analysis, optimal cutoff values of these parameters in the prediction of postoperative VTE were identified (Table 3). Moreover, based on the Hosmer-Lemeshow test, our calibration curve results indicated that D-Dimer preoperative (*P* = 0.565) and postoperative day 3 (*P* = 0.439), MPV postoperative day 3 (*P* = 0.319) and day 5 (*P* = 0.172), and TEG-MA postoperative day 1 (*P* = 0.840), day 3 (*P* = 0.162), and day 5 (*P* = 0.459) all have good calibration (Fig. 3).

**Table 3:** ROC curve related parameters

	ROC curve area	Hazard ratio (95% CI)	Youden index	Cutoff point
Pre-D-Dimer	0.758	0.622–0.895	0.422	493.85
D3-D-Dimer	0.827	0.680–0.974	0.619	1212.25
D3-MPV	0.797	0.626–0.967	0.604	11.35
D5-MPV	0.878	0.760–0.996	0.742	11.00
D1-MA	0.773	0.677–0.869	0.511	69.25
D3-MA	0.849	0.689-1.000	0.706	73.05
D5-MA	0.807	0.633–0.981	0.655	73.15

CI: confidence in interval, MPV: mean platelet volume, MA: maximum amplitude of thromboelastography

**Figure**:

## Discussion

In this study, we tried to investigate whether relevant biochemical parameters are associated with the occurrence of VTE after minimally invasive colorectal cancer surgery in a retrospective observational study. Our results showed that increased D-Dimer, MPV, and TEG-MA were positively associated with postoperative VTE in patients undergoing minimally invasive surgery for colorectal cancer at specific times in the perioperative period. Both our ROC curves and calibration curves confirmed the association. These results indicated that D-Dimer, MPV, and TEG-MA may be potential biochemical parameters to predict the occurrence of VTE after minimally invasive surgery for colorectal cancer.

In recent years, minimally invasive surgery has become more common and is considered to be the direction of surgical development [18]. In this study, our minimally invasive surgery included laparoscopic and robotic surgery, and 97 and 52 CRC patients were collected in the two groups, respectively. Regardless of the type of surgery performed, the potential risk for postoperative complications exists. In CRC surgery, in addition to complications such as anastomotic dehiscence and wound infection, the occurrence of postoperative VTE is equally life-threatening to patients. VTE is a serious complication of surgical procedures and is a preventable cause of death in patients hospitalized for surgery [19, 20]. Compared to non-VTE patients, VTE patients have significantly more complexity and financial burden of treatment [21]. Guidelines recommend antithrombotic therapy after surgery [22], and antithrombotic prophylaxis has been reported to reduce the incidence of venous thromboembolism by approximately 70% [23]. Despite the use of antithrombotic prophylaxis, the incidence of symptomatic venous thromboembolism within one month after major cancer surgery was reported to be 2.1% [24]. For CRC surgery, the risk of developing a significantly symptomatic VTE has been previously reported to be approximately 2–4% [25, 26]. In our study, the cumulative incidence of VTE after minimally invasive colorectal cancer surgery was 8.1%, and we not only collected patients with symptomatic VTE but also included patients with asymptomatic VTE. In our study center, 88 patients underwent rectal cancer surgery (59.1%), although our study showed no statistically significant difference between the type of resection and the occurrence of VTE. However, the rectal surgical procedure was performed in the lithotomy position, and previous studies have shown that surgery in the lithotomy position is a potential risk for venous thrombosis [27]. Furthermore, we found that the occurrence of postoperative VTE was associated with increased ASA scores, with the incidence of postoperative VTE occurring in ASA scores 2 and 3 being 3.4% and 14.8%, respectively.

Aside from antithrombotic therapy, it also appears essential to predict the risk of venous thromboembolism in cancer patients and to provide targeted treatment. Tian et al. found that after performing pulmonary surgery, patients with postoperative VTE had significantly higher D-Dimer preoperatively and at postoperative day 1 and day 3 than non-VTE patients [28]. In breast cancer, it has been shown that D-Dimer is significantly increased in patients with DVT after radical breast cancer surgery [29]. In our study, D-Dimer was significantly higher in VTE patients than in non-VTE patients at preoperative and postoperative day 3, which were 550.18 ± 357.71 vs. 279.58 ± 225.54, *P* = 0.028; 1564.77 ± 672.11 vs. 809.31 ± 443.35, *P* = 0.014, respectively. Previous studies have shown that MPV was an independent predictor of DVT and was significantly elevated in patients with DVT [30, 31]. Our results indicated that MPV was higher in VTE patients compared to non-VTE patients at postoperative day 3 and day 5, which were 12.40 ± 1.85 vs. 10.58 ± 0.93, *P* < 0.001, and 12.61 ± 1.43 vs. 10.56 ± 1.12, *P* = 0.046, respectively. Moreover, we also found that TEG-MA was also higher in VTE patients than in non-VTE patients at postoperative day 1, day 3, and day 5 (70.75 ± 0.90 vs. 68.81 ± 2.39, *P* = 0.015; 73.78 ± 3.90 vs. 70.21 ± 2.16, *P* = 0.021; 74.27 ± 2.69 vs. 71.24 ± 1.84, *P* = 0.029). Therefore, D-Dimer, MPV, and TEG-MA may play an important role in the prediction of postoperative VTE during specific periods of the perioperative period.

Then, we constructed ROC curves to assess the predictive power of D-Dimer, MPV, and TEG-MA for postoperative VTE occurrence. Our results showed that the area under the ROC curve for D-Dimer was 0.758 and 0.827 at preoperative and postoperative day 3, respectively. The area under the ROC curve for MPV was 0.797 and 0.878 at postoperative day 3 and day 5. The area under the ROC curve for TEG-MA at postoperative day 1, day 3, and day 5 were 0.773, 0.849, and 0.807. The results of ROC analysis showed that D-Dimer at preoperative, postoperative day 3, MPV at postoperative day 3, day 5, and TEG-MA at postoperative day 1, day 3, and day 5 were all discriminatory for postoperative VTE prediction. Finally, we constructed calibration curves based on the Hosmer-Lemeshow test. Our calibration curves showed good calibration of D-Dimer at preoperative, postoperative day 3, MPV at postoperative day 3, day 5, and TEG-MA at postoperative day 1, day 3, and day 5 for predicting VTE (all *P*-values > 0.05).

There are several limitations in our study. First, the sample size of this study is small and still needs to be confirmed by a large sample of multicenter studies. Second, the results of this study were only applicable to Chinese patients who underwent minimally invasive colorectal cancer surgery, and further studies are needed for other ethnic groups and surgical modalities. Third, our study excluded patients with a previous history of thromboembolism but failed to exclude patients who initially had subclinical VTE before surgery. Further studies are still needed for this group of patients.

In conclusion, D-Dimer at preoperative and postoperative day 3, MPV at postoperative day 3 and day 5, and TEG-MA at postoperative day 1, day 3, and day 5 may predict postoperative VTE in patients undergoing minimally invasive colorectal cancer surgery. Early prediction and diagnosis of postoperative VTE may provide important guidance for high-risk patients.

## Data Availability

The data sets generated and analyzed during the current study are not publicly available due to restrictions on ethical approvals involving patient data and anonymity but can be obtained from the corresponding author at reasonable request.
